# BNIP3/BNIP3L-Dependent Mitophagy Protects Against Hippocampal Neuronal Damage and Apoptosis in a Model of Vascular Dementia

**DOI:** 10.3390/cells15070585

**Published:** 2026-03-25

**Authors:** Yujiao Wang, Daojun Xie, Shijia Ma, Yuhe Wang, Chengcheng Zhang, Zhuyue Chen

**Affiliations:** The First Clinical Medical College of Anhui University of Chinese Medicine, Hefei 230036, China; 2023501104050@stu.ahtcm.edu.cn (Y.W.);

**Keywords:** vascular dementia, mitophagy, apoptosis, BNIP3, BNIP3L, ROS

## Abstract

**Highlights:**

**What are the main findings?**

- BNIP3 and BNIP3L are significantly downregulated under chronic cerebral hypoperfusion, leading to impaired receptor-mediated mitophagy and neuronal apoptosis. Overexpression of BNIP3 or BNIP3L restores mitophagic flux, reduces oxidative stress, and attenuates apoptosis in an autophagy-dependent manner.

**What are the implications of the main findings?**

- This study identifies BNIP3/BNIP3L as critical regulators of mitochondrial quality control in vascular dementia and highlights their potential as therapeutic targets for mitigating vascular cognitive decline.

**Abstract:**

Mitophagy serves as an essential quality control mechanism that maintains mitochondrial homeostasis through selective autophagic clearance of damaged organelles. Vascular dementia (VD) has been increasingly associated with mitophagy dysregulation in recent studies. However, the precise molecular mechanisms underlying mitophagy’s involvement in VD pathogenesis remain poorly characterized. To elucidate the role of mitophagy in VD, we systematically examined the expression of key mitophagy pathways in hippocampal neurons of bilateral common carotid artery occlusion (BCCAO) rats and in oxygen–glucose deprivation (OGD)-treated HT22 cells. Intriguingly, under autophagy-deficient conditions, both BNIP3 and BNIP3L were markedly downregulated, whereas FUNDC1 expression increased; PINK1/Parkin levels remained unaltered. To further dissect the functional contributions of BNIP3 and BNIP3L, we administered the mitochondrial fission inhibitor Mdivi-1 to BCCAO model rats. Histopathological analysis revealed pronounced neuronal damage and apoptosis in the hippocampal region, which was further exacerbated upon Mdivi-1 treatment. In vitro, BNIP3 silencing significantly compromised cell viability, elevated reactive oxygen species (ROS) accumulation, disrupted mitochondrial membrane potential (ΔΨm), suppressed mitophagy, and increased apoptotic rates. Conversely, BNIP3 overexpression reversed these detrimental effects. Notably, treatment with the autophagy inhibitor 3-methyladenine (3-MA) diminished LC3B-Tomm20 colocalization and intensified apoptosis, reinforcing the critical role of BNIP3-mediated mitophagy in neuronal survival. Similarly, BNIP3L overexpression enhanced cell viability, attenuated ROS production, restored ΔΨm, and mitigated apoptosis, while 3-MA treatment again impaired mitophagic flux and worsened cell death. Collectively, these findings underscore the critical and distinct roles of BNIP3 and BNIP3L in maintaining mitochondrial homeostasis and neuronal survival under ischemic conditions.

## 1. Introduction

Dementia represents a growing global health challenge, currently affecting approximately 50 million individuals worldwide, with projections suggesting a tripling of this burden by 2050 [1]. Among dementia subtypes, vascular dementia (VD) ranks as the second most common form after Alzheimer’s disease (AD) [2], accounting for approximately 20% of pure dementia cases, with an additional 20% contributing to mixed dementia pathology. VD encompasses a spectrum of cerebrovascular-related cognitive disorders characterized by distinct clinical trajectories. Epidemiological data reveal that VD patients typically experience symptom onset at 67.5 ± 7.2 years, receive diagnosis at 73.5 ± 7.0 years, and demonstrate significantly shorter post-diagnosis survival (3.2 ± 1.4 years) compared to other major dementia subtypes (AD: 5.8 ± 2.0 years; Lewy body dementia: 4.7 ± 1.8 years; frontotemporal dementia: 4.9 ± 2.2 years) [3]. This accelerated disease progression, coupled with a mean age of death at 77.0 ± 6.9 years, underscores the urgent need to elucidate pathogenic mechanisms and identify novel therapeutic targets for VD, with profound implications for clinical practice.

The pathogenesis of VD involves multifactorial mechanisms that vary by clinical subtype, though shared pathological features exist [4]. A key contributor is mitochondrial dysfunction, where impaired mitophagy—a critical quality-control process for eliminating damaged mitochondria—disrupts cellular homeostasis and exacerbates VD progression [5]. Ischemia-hypoxia triggers energy failure, reducing ATP synthesis while elevating reactive oxygen/nitrogen species (ROS/RNS) [6,7]. Chronic cerebral hypoperfusion (CCH) in VD induces mitochondrial dysfunction in vivo, a process characterized by membrane potential collapse, oxidative stress, and can involve excitotoxicity, ultimately leading to the activation of caspase-3/BAX-mediated apoptotic pathways [4,5]. The bilateral common carotid artery occlusion (BCCAO) model is a well-established method to induce chronic cerebral hypoperfusion, a core mechanism in subcortical ischemic vascular dementia, and has been widely used to study associated mitophagy impairment [8,9]. This cascade disturbs calcium handling, redox balance, and autophagy, impairing neurotransmission, axonal transport, and myelination [10]. Collectively, these deficits promote neuronal death, accelerating cerebrovascular injury and cognitive decline.

The PINK1-Parkin pathway, activated by mitochondrial depolarization, has been implicated in neuroprotection, yet its physiological relevance in basal mitophagy remains debated [11,12,13]. While this pathway primarily responds to acute mitochondrial insults [14,15], receptor-mediated mitophagy—particularly through BNIP3, BNIP3L, and FUNDC1—dominates under chronic conditions, especially in energy-demanding tissues like the brain [16]. Notably, Alzheimer’s and Parkinson’s disease patients exhibit suppressed BNIP3/BNIP3L- and FUNDC1-dependent mitophagy without PINK1-Parkin alterations [17,18,19,20,21,22], highlighting the critical role of receptor-mediated pathways in chronic stress adaptation [12,13]. BNIP3, an outer mitochondrial membrane (OMM) protein of the BH3-only Bcl-2 family, directly binds LC3 to trigger PARK2-independent mitophagy [23,24]. Its homolog BNIP3L (NIX) similarly regulates mitochondrial quality control. Emerging evidence suggests BNIP3/BNIP3L not only sustains basal mitophagy but also compensates for Parkin deficiency, underscoring their dual role in mitochondrial homeostasis [24].

This study employed BCCAO rats and oxygen–glucose deprivation (OGD)-treated HT22 cells, well-established models of VD, to elucidate the mechanistic link between CCH and mitophagy dysregulation. Through integrated in vivo and in vitro approaches, we systematically investigated how BNIP3/BNIP3L-dependent mitophagy impairment contributes to the disruption of complete autophagic flux, neuronal apoptosis, and ultimately, CCH-induced cognitive deficits.

## 2. Materials and Methods

### 2.1. Antibodies

The following primary antibodies were used: BNIP3 (Abways, CY6771, Shanghai, China), BNIP3L (Abways, CY6906), β-actin (Affinity, Cincinnati, OH, USA, AF7018), Tomm20 (Abways, CY5527), Beclin1 (Abways, CY5092), P62 (Abways, CY5546), LC3B (Abways, CY5992), PINK1 (Proteintech, 23274-1-AP, Wuhan, China), Parkin (Abways, CY6641), FUNDC1 (Biodragon, BD-PT5658, Suzhou, China), Bcl2 (Abways, CY6717), BAX (Abways, CY5059), Caspase3 (Abways, CY5501), GAPDH (Abways, AB0037), Tubulin (Boster, A01857-1, Wuhan, China).

### 2.2. Chemicals and Reagents

Opti-MEMTM (GIBCO, Pittsburgh, PA, USA, 11058021), Dulbecco’s modified Eagle’s medium (DMEM, GIBCO, 11966025), Fetal bovine serum (FBS, Servicebio, G8003, Wuhan, China), Penicillin and streptomycin (Sparkjade, CM0001, Jinan, China), PhosSTOP (phosphatase inhibitor, Beyotime, P1081, Shanghai, China), Protease Inhibitor Cocktail (Sparkjade, Jinan, China, SJ-MK0001), Phenylmethanesulfonyl fluoride (PMSF, Sparkjade, EA0005), and Lipo3000 (Thermo, L3000-015, Waltham, MA, USA), Mdivi-1 (Sparkjade, SJ-MX0169), 3-MA (Sparkjade, SJ-MN0032), Bafilomycin A1 (Sparkjade, SJ-MA0035).

### 2.3. Animals

Male Sprague-Dawley rats (*n* = 45; 8 weeks old; 200 ± 10 g) were obtained from a certified vendor (Qualified Number: SCXK (Liao) 2020–0002). Animals were housed under specific pathogen-free (SPF) conditions with controlled temperature (22–25 °C), humidity (60%), and a 12-h light/dark cycle, with ad libitum access to food and water. All procedures complied with the National Institutes of Health (NIH) Guide for the Care and Use of Laboratory Animals and were approved by the Experimental Animal Ethics Committee of Anhui University of Chinese Medicine (Ethics Certificate Number: AZYFY-2024-1002; Approval Date: 30 July 2024).

### 2.4. Establishment of a VD Model and Drug Treatment

The rat model of CCH was established by BCCAO [25,26]. Briefly, rats were anesthetized, and the common carotid arteries (CCA) were ligated with silk sutures after midline neck incision. Sham-operated rats underwent identical procedures without CCA ligation.

Forty-five rats were randomly divided into three groups: the sham operation group, the BCCAO model group, and the BCCAO+ Mdivi-1 (a mitochondrial fission inhibitor) group. The Mdivi-1 (1.2 mg/Kg/day) [27] group was dissolved in a solvent of 10% DMSO + 5% Tween 80 + 85% normal saline and was intraperitoneally injected once a day for 4 consecutive weeks. The Sham group and the BCCAO group were given the same solvent for abdominal cavity annotation every day.

### 2.5. Cerebral Blood Flow (CBF) Measurement

According to the previous study [28], Regional CBF was assessed by laser speckle contrast imaging (SIM BFI HR Pro, Wuhan Xunwei Optoelectronic Technology Co., Wuhan, China) at four time points: preoperative baseline, 24 h post-operation, 1 week post-operation, 4 weeks post-operation. Three rats per group were analyzed at each time point.

### 2.6. Morris Water Maze (MWM) Test

As previously described [29], spatial learning and memory in rats were assessed using the MWM at 4 weeks after surgery. The protocol consisted of a 5-day acquisition training phase followed by a probe trial. During the training phase (days 1–5), rats underwent four trials per day to locate a hidden platform, and the escape latency was recorded for each trial to evaluate spatial learning. 24 h after the last training session, a single probe trial was conducted with the platform removed. Spatial memory retention was assessed by measuring both the time spent in the target quadrant and the number of crossings over the former platform location. All behavioral analyses used *n* = 6 rats per group.

### 2.7. Histologic Examination

After rats were deeply anesthetized with an intraperitoneal injection of 2.5% tribromoethanol, they were euthanized via transcardial perfusion with PBS or 4% paraformaldehyde (PFA) for tissue fixation. Brains were removed, post-fixed in 4% PFA at 4 °C overnight, dehydrated, paraffin-embedded, and coronally sectioned at 4 µm. Coronal sections containing the hippocampal formation were selected for subsequent histological analyses (*n* = 3 rats per group).

### 2.8. Hematoxylin and Eosin (HE) Staining

Fresh brain tissue samples randomly selected from each group were fixed in 4% paraformaldehyde, embedded in paraffin, and sectioned at 4 μm thickness. Following deparaffinization and dehydration, the sections were stained with HE. Excess dye was removed by washing, and the sections were subsequently dehydrated through a graded alcohol series, cleared in xylene, and mounted with a resinous medium for microscopic analysis (*n* = 3 rats per group).

### 2.9. Nissl Staining

Nissl staining was performed using a commercial Nissl stain kit in accordance with the manufacturer’s instructions. Representative images of the cortical and hippocampal regions were acquired using light microscopy at magnifications of ×200 and ×400 (*n* = 3 rats per group).

### 2.10. TUNEL Staining

Apoptotic cells were detected using a commercial TUNEL Apoptosis Assay Kit (Elabscience, E-CK-A331, Wuhan, China). Following dewaxing, tissue sections were treated with proteinase K and incubated with the TUNEL reaction mixture. Cell nuclei were counterstained with DAPI (4′,6-diamidino-2-phenylindole). Subsequently, sections were dehydrated, cleared in xylene, and mounted with neutral gum. Apoptotic cells were identified by characteristic brown staining under light microscopy (*n* = 3 rats per group).

### 2.11. Immunofluorescence Double-Labeling

Following dewaxing, antigen retrieval, and blocking of endogenous enzymes and non-specific binding sites, brain sections were incubated overnight at 4 °C with primary antibodies against Tomm20 (1:200 dilution, HUABIO, Woburn, MA, USA, ET1609-25) and LC3B (1:200 dilution, HUABIO, ET1701-65). After three 10-min PBS washes, sections were incubated with appropriate secondary antibodies for 1 h at 37 °C. Following another series of PBS washes (3 × 10 min), nuclei were counterstained with DAPI (4′,6-diamidino-2-phenylindole) for 30 min at 37 °C. Finally, sections were mounted with anti-fade mounting medium. Fluorescence images were captured using a Leica fluorescence microscope (Leica Microsystems, Wetzlar, Germany), and colocalization analysis of LC3B and Tomm20 signals was performed using the JACoP plugin in ImageJ software (version 1.54p, *n* = 3 per group).

### 2.12. Transmission Electron Microscope (TEM) Test

After completion of the Morris water maze test, the hippocampal CA1 region was carefully dissected and sectioned into 1.0 mm^3^ cubic tissue blocks, which were immediately fixed in 1% osmium tetroxide. Following standard dehydration through a graded ethanol series, the samples underwent epoxy resin permeation, embedding, and polymerization. Ultrathin sections (70 nm) were prepared using an ultramicrotome and double-stained with 2% uranyl acetate in saturated alcohol solution followed by 2.6% lead citrate. The prepared sections were then examined using a Hitachi HT7700 transmission electron microscope (Hitachi High-Tech Corporation, Tokyo, Japan; *n* = 3 rats/group).

### 2.13. Western Blot Analysis

According to previous study [30], total protein was extracted from the right hippocampus using RIPA lysis buffer. Protein samples (30 μg per lane) were separated by SDS-PAGE and electrophoretically transferred to PVDF membranes. The membranes were then blocked and incubated overnight at 4 °C with the following primary antibodies: anti-P62 (1:5000), anti-Beclin1 (1:5000), anti-LC3B (1:2000), anti-Tomm20 (1:2000), anti-PINK1 (1:1000), anti-Parkin (1:2000), anti-BNIP3 (1:20,000), anti-BNIP3L (1:2000), anti-FUNDC1 (1:2000), anti-GAPDH (1:10,000), anti-Tubulin (1:10,000), and anti-β-actin (1:5000). After washing, membranes were incubated with appropriate HRP-conjugated secondary antibodies. Protein bands were visualized using enhanced chemiluminescence (ECL) reagent and quantified using ImageJ software (version 1.54p).

### 2.14. Cell Culture and Transfection

HT22 cells (Servicebio, STCC20011P) were cultured in high-glucose DMEM supplemented with 10% fetal bovine serum (FBS) and 50 μg/mL penicillin/streptomycin at 37 °C in a humidified 5% CO_2_ incubator [31]. At 50–60% confluence, cells were transfected using Lipofectamine 3000 reagent [32] with either: (1) mouse BNIP3 siRNA (5′-CAUGAAUCUGGACGAAGUA-3′, 50 pmol/well in 6-well plates; Fenghuishengwu, Changsha, China) for gene silencing, or (2) BNIP3 pcDNA3.1(+) or BNIP3L pcDNA3.1(+) plasmids (Fenghuishengwu) for gene overexpression. Control groups received equivalent amounts of negative control siRNA (NC siRNA) or empty pcDNA3.1(+) vector (NC pcDNA3.1(+)). Transfection efficiency was evaluated by WB analysis 48 h post-transfection following the manufacturer’s protocol.

### 2.15. OGD Model

For OGD model establishment, HT22 cells were seeded in 6-well plates at 30% confluence and maintained under standard culture conditions (5% CO_2_, 37 °C) for 24 h. Subsequently, the culture medium was replaced with glucose-free DMEM supplemented with 10% fetal bovine serum, and cells were transferred to a tri-gas incubator (1% O_2_, 5% CO_2_, 94% N_2_) for 24 h to induce ischemic-like conditions [33]. Immediately following the 24-h OGD period, cells were processed for subsequent assays, including the assessment of cell viability (CCK-8), intracellular ROS levels, mitochondrial membrane potential (JC-1), and apoptosis.

### 2.16. CCK-8 Assay

Following completion of cell modeling and drug treatments, the culture medium was carefully aspirated from each well. Fresh medium containing 10% CCK-8 reagent (100 μL/well) was then added. The plate was protected from light by wrapping with aluminum foil and incubated under standard conditions (37 °C, 5% CO_2_) for 30 min. Absorbance was subsequently measured at 450 nm using a microplate reader.

### 2.17. Measurement of HT22 Apoptosis

Apoptosis was evaluated using a commercial apoptosis detection kit (Elabscience, AK16006) combined with flow cytometric analysis. Following experimental treatments, cells were fixed with 4% paraformaldehyde (PFA) for nuclear staining. For morphological assessment, fixed cells were incubated with Hoechst 33258 (5 μg/mL) for 15 min at room temperature protected from light, and apoptotic nuclei (condensed or fragmented chromatin) were visualized using an inverted fluorescence microscope (Olympus, Tokyo, Japan).

For quantitative analysis, harvested cells were resuspended in binding buffer and sequentially stained with 5 μL Annexin V-FITC and 10 μL propidium iodide (PI) (20 μg/mL) for 15 min at room temperature in the dark. Apoptotic cell populations were then quantitatively analyzed using a NovoCyte flow cytometer (Anjielun, San Diego, CA, USA) with NovoExpress software (version 2.2.0). Early apoptotic (Annexin V+/PI−), late apoptotic (Annexin V+/PI+), and necrotic (Annexin V−/PI+) populations were distinguished based on fluorescence intensity.

### 2.18. ROS Determination

Following experimental treatments, cells were harvested by trypsinization and the reaction was quenched with complete culture medium. The cell suspension was gently pipetted to ensure single-cell dispersion, then washed and resuspended in serum-free medium. After performing cell counting using a hemocytometer, cells were incubated with 10 μM DCFH-DA fluorescent probe in serum-free medium at 37 °C for 30 min in the dark. Following incubation, cells were washed twice with PBS to remove excess probe, and intracellular ROS levels were immediately quantified by flow cytometry (NovoCyte, ACEA Biosciences, San Diego, CA, USA) using 488 nm excitation and 530 nm emission settings.

### 2.19. Detection of Mitochondrial Membrane Potential (MMP)

According to the manufacturer’s instructions (Beyotime, C2006), JC-1 could aggregate and fluoresce red in healthy mitochondria, whereas it fluoresces green in depolarized mitochondria. After treatment with JC-1 detection reagent, washing, centrifu-gation and resuspension, flow cytometry was used to obtain the mean fluorescence intensity. The entire experiment was done in a dark environment.

### 2.20. Statistical Analysis

The experimental data are presented as mean ± standard deviation (SD) throughout the manuscript. Statistical analyses were conducted using GraphPad Prism software (version 8.0). For multi-group comparisons, we performed one-way analysis of variance (ANOVA) followed by Tukey’s post hoc analysis for pairwise comparisons. Specifically for the laser speckle imaging-derived blood flow measurements, between-group differences were assessed using unpaired Student’s *t*-tests. A uniform significance threshold of *p* < 0.05 was applied to all statistical evaluations, with exact *p*-values reported for transparency.

## 3. Results

### 3.1. Successful Establishment of CCH Model with Associated Cognitive Deficits

**Figure 1:**
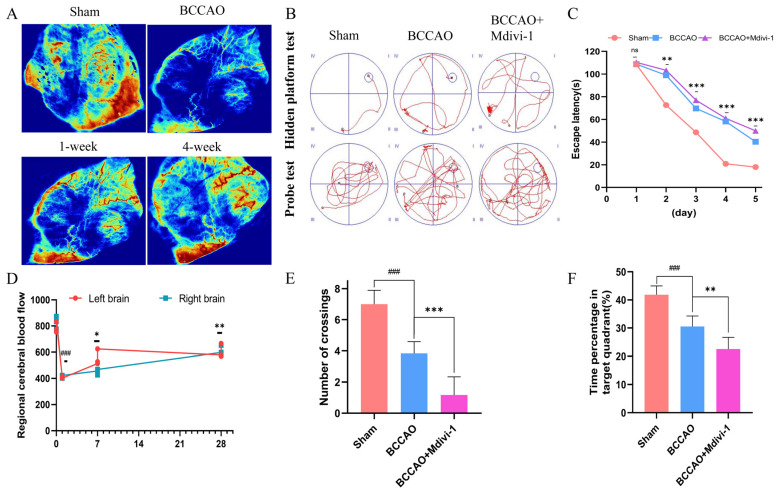
BCCAO induces CCH and cognitive deficits, exacerbated by mitophagy inhibition. (**A**) Representative CBF heatmaps. (**B**) The representative trajectory map of the rat MWM test. (**C**) MWM escape latency (Days 1–5) showing spatial learning deficits in BCCAO rats, worsened by Mdivi-1 (*n* = 6 rats per group). ** *p* < 0.01 vs. sham, *** *p* < 0.001 vs. sham. (**D**) Longitudinal CBF changes (laser speckle imaging) in sham and BCCAO rats at baseline, 24 h, 1 week, and 4 weeks post-surgery (*n* = 3 rats/group). ^###^ *p* < 0.001 vs. sham, * *p* < 0.05 vs. 24 h, ** *p* < 0.01 vs. 24 h. (**E**,**F**) Memory retrieval deficits: (**E**) Platform crossings and (**F**) target quadrant occupancy in probe trial. ^###^ *p* < 0.001 vs. sham; ** *p* < 0.01 and *** *p* < 0.001 vs. BCCAO. Data = mean ± SD; *n* = 6 rats/group.

The BCCAO model is a classic animal model used to mimic CCH. To validate this model, we longitudinally monitored CBF at baseline and 24 h, 1 week, and 4 weeks post-surgery. The CBF level precipitously dropped to <50% of baseline values at 24 h, partially recovered at 1 week, and stabilized at 70% of baseline by 4 weeks (Figure 1A,D), thereby confirming sustained hypoperfusion characteristic of CCH.

Consistent with CBF alterations, BCCAO rats displayed pronounced learning and memory impairments (Figure 1B). As shown in Figure 1C, spatial learning deficits were evidenced by increased escape latency in the MWM. Memory retrieval impairment was demonstrated through reduced platform crossings (Figure 1E) and shorter target quadrant occupancy (Figure 1F).

Notably, four-week Mdivi-1 treatment (a mitochondrial fission inhibitor) exacerbated these cognitive deficits, demonstrating that suppression of mitochondrial autophagy aggravates CCH-induced neurodegeneration (Figure 1B,C,E,F).

### 3.2. BNIP3/BNIP3L Pathway Deficiency Is a Critical Driver of CCH Pathogenesis

**Figure 2:**
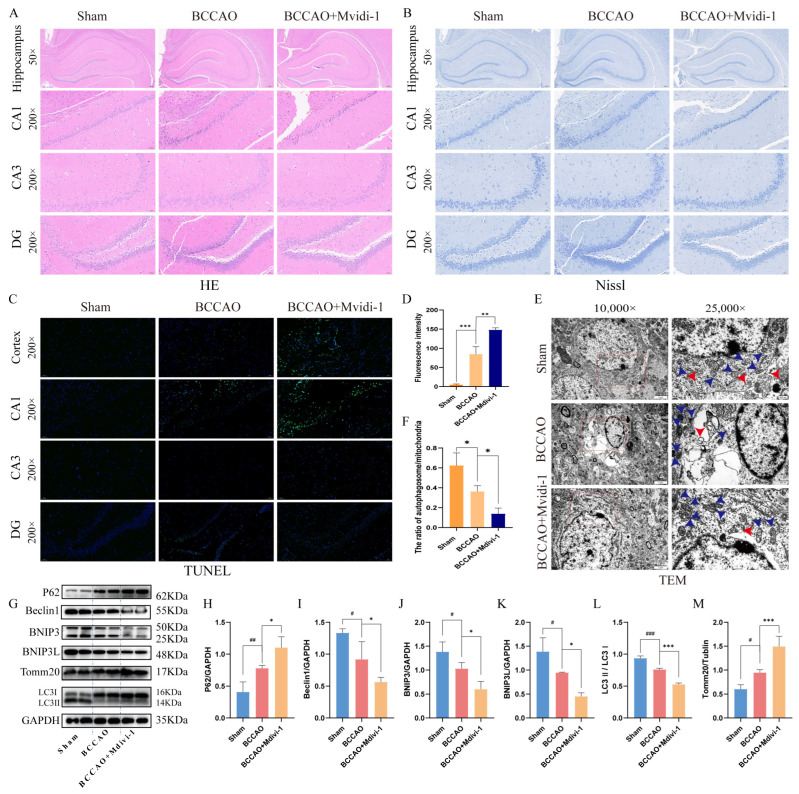
CCH induces neuronal damage and autophagy suppression via BNIP3/BNIP3L pathway dysfunction. (**A**) Representative images of hippocampal neuronal histopathology in HE staining (scale bar: 50 μm). (**B**) Representative images of hippocampal neuronal histopathology in Nissl staining (scale bar: 200 or 50 μm). (**C**) TUNEL staining showing apoptotic neurons (brown nuclei) in the cortex and hippocampal CA1, CA3, DG region (scale bar: 50 μm). (**D**) Quantification of TUNEL-positive cells in CA1 region (** *p* < 0.01, *** *p* < 0.001; *n* = 3 rats/group). (**E**) Electron micrographs of hippocampal neurons. BCCAO group showed mitochondrial vacuolation, nuclear membrane rupture (blue arrow), and reduced number of autophagic structures (red arrow) (scale bar: 2 μm or 500 nm). (**F**) The ratio of autophagic structures (including autophagosomes and autolysosomes) to mitochondria across multiple fields from each group (* *p* < 0.05, *n* = 3 rats/group). (**G**) The representative strip of WB. (**H**–**M**) WB analysis of autophagy and mitophagy markers (^#^ *p* < 0.05, ^##^ *p* < 0.01, ^###^ *p* < 0.001 vs. sham; * *p* < 0.05 and *** *p* < 0.001 vs. BCCAO. Data = mean ± SD; *n* = 4 repetitions/group).

Histopathological analysis revealed marked neuropathological alterations in BCCAO rats compared to sham controls. Figure 2A showed neuronal degeneration, including pyknotic nuclei, cellular atrophy, disorganized neuronal architecture, and significant cell loss, along with reduced hippocampal Nissl bodies (Figure 2B). Four-week Mdivi-1 treatment significantly aggravated these pathological changes. The number of apoptotic neurons in the cortical and hippocampal CA1 regions increased significantly in BCCAO models, and Mdivi-1 treatment aggravated apoptosis (Figure 2C,D). In addition, the BCCAO group showed impaired mitochondrial ultrastructure, as shown by mitochondrial deformation, reduction and disappearance of mitochondrial ridge structure, mitochondrial vacuolization, and nuclear membrane disruption (Figure 2E, blue arrow). Moreover, reduced number of autophagic structures (red arrows) in the BCCAO group, including double-membrane autophagosomes, many of which are in proximity to or appear to engulf cellular organelles (Figure 2E, red arrow). This trend was further aggravated after Mdivi-1 treatment (Figure 2F).

WB analysis demonstrated autophagy suppression in BCCAO rats (Figure 2G), characterized by: (a) accumulated autophagic substrates (increased p62; Figure 2H); (b) impaired autophagosome formation (reduced Beclin1 and LC3-II/LC3-I ratio, respectively; Figure 2I,L); (c) dysregulated mitophagy markers, with significant downregulation of BNIP3/BNIP3L (Figure 2J,K) and upregulated FUNDC1 (Figure S1C,F), but unaltered PINK1/Parkin levels (Figure S1A,B,D,E); and (d) mitochondrial accumulation (elevated Tomm20; Figure 2M). Notably, Mdivi-1 treatment exacerbated mitochondrial autophagy suppression, amplifying mitochondrial overload (Figure 2G–M) and neurodegeneration, suggesting BNIP3/BNIP3L pathway deficiency as a critical driver of CCH pathogenesis.

### 3.3. OGD Recapitulates Mitophagy Dysregulation in HT22 Cells

**Figure 3:**
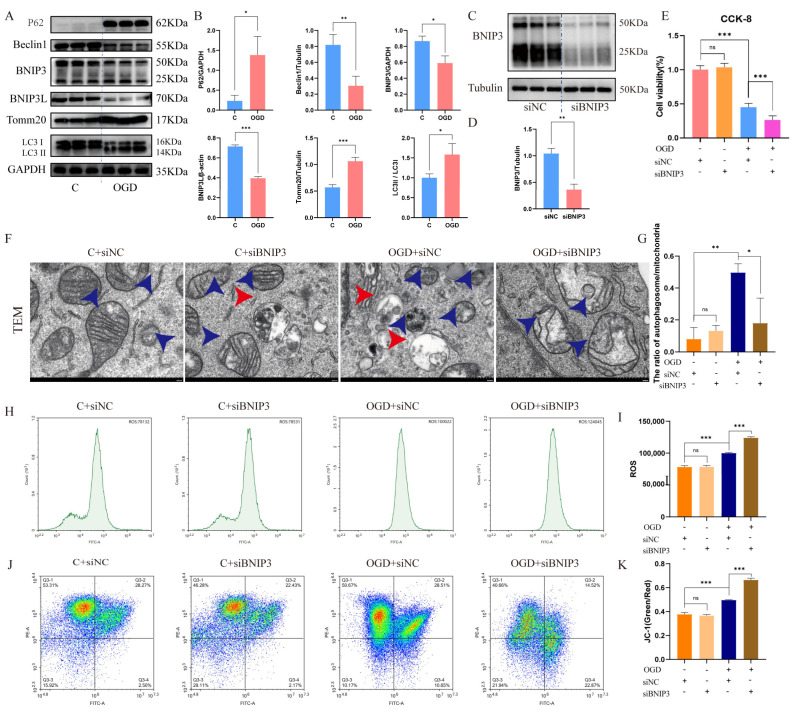
BNIP3 knockdown exacerbates OGD-induced neuronal injury by impairing mitophagy and mitochondrial function. (**A**) Representative blots showing p62, LC3-I/II, Beclin1, Tomm20, BNIP3, and BNIP3L expression. (**B**) Quantification of protein levels normalized to β-actin, GAPDH, or Tubulin. (* *p* < 0.05, ** *p* < 0.01, *** *p* < 0.001 vs. control; Data = mean ± SD; *n* = 3 repetitions/group). (**C**) Representative WB of BNIP3 after siRNA transfection. (**D**) Quantification of BNIP3 protein levels (** *p* < 0.01 vs. siNC; Data = mean ± SD; *n* = 3 repetitions/group). (**E**) Cell viability (CCK-8 assay) in HT22 neurons under OGD with/without siBNIP3 (*** *p* < 0.001; Data = mean ± SD; *n* = 3 repetitions/group). (**F**) Representative TEM images of each group in HT22 cells (scale bar: 500 nm). (**G**) The ratio of autophagic structures to mitochondria across multiple fields from each group (* *p* < 0.05, ** *p* < 0.01, *n* = 3 repetitions /group). (**H**) Representative flow cytometry images of each group. (**I**) Quantification of ROS intensity (*** *p* < 0.001; Data = mean ± SD; *n* = 3 repetitions/group). (**J**) JC-1 aggregates (red, healthy) and monomers (green, depolarized). (**K**) Quantification of JC-1 green/red ratio (*** *p* < 0.001; Data = mean ± SD; *n* = 3 repetitions/group).

To elucidate the cellular mechanisms underlying CCH-induced mitophagy impairment, we established an in vitro OGD model in HT22 hippocampal neurons. Following 24 h OGD exposure, we observed (Figure 3A,B) significant accumulation of p62 and an increased LC3-II/LC3-I ratio, accompanied by reduced Beclin1 expression, suggesting complete autophagic flux blockade. Additionally, elevated Tomm20 levels with concurrent downregulation of BNIP3/BNIP3L (Figure 3A,B) indicated impaired mitochondrial clearance. FUNDC1 upregulation without alterations in PINK1/Parkin (Figure S1G–L) confirmed the specificity of the BNIP3/BNIP3L pathway in vitro, consistent with in vivo findings. These results demonstrate that OGD-induced mitophagy suppression is primarily mediated through BNIP3/BNIP3L pathway inhibition, leading to defective mitochondrial turnover and abnormal organelle accumulation.

### 3.4. BNIP3 Knockdown Exacerbates OGD-Induced Mitochondrial Dysfunction

To establish causality, we performed BNIP3 silencing in HT22 cells (siRNA efficiency > 70%; Figure 3C,D). Cell viability assessment (CCK-8 assay) revealed that BNIP3 silencing alone did not affect viability under normoxic conditions. However, OGD treatment significantly reduced viability versus controls, and siBNIP3 further potentiated this effect (Figure 3E).

In addition, TEM of HT22 cells showed that under normoxic conditions, the mitochondrial structure was clear with intact cristae and no swelling; silencing BNIP3 under normoxia had no effect on mitochondrial morphology. In contrast, OGD treatment resulted in blurred mitochondrial structure, shrinkage and loss of cristae, accompanied by an increase in mitophagosomes. However, under OGD conditions, silencing BNIP3 led to marked mitochondrial swelling, structural disruption, and a significant reduction in mitophagosomes (Figure 3F,G).

Similarly, while siBNIP3 had no effect on ROS levels in normoxia, OGD treatment significantly increased ROS production. BNIP3 knockdown in OGD-treated cells caused a further ROS elevation (Figure 3H,I).

Mitochondrial membrane potential assessment yielded consistent results: siBNIP3 alone did not alter ΔΨm, whereas OGD increased the JC-1 green/red ratio. Combined OGD and siBNIP3 treatment exacerbated ΔΨm loss (Figure 3J,K).

Collectively, these data indicate that BNIP3 knockdown exacerbates OGD-induced neuronal injury by impairing mitophagy and amplifying mitochondrial dysfunction.

### 3.5. BNIP3 Deficiency Impairs Mitophagic Flux and Exacerbates Apoptotic Signaling

**Figure 4:**
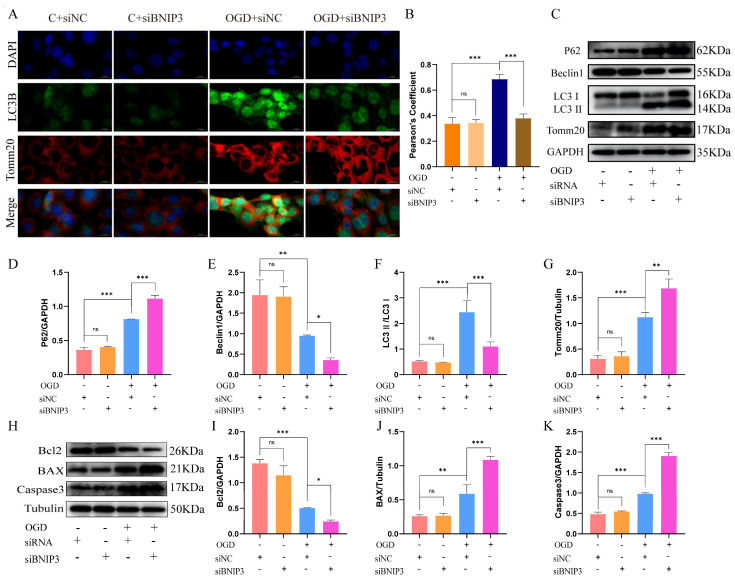
BNIP3 deficiency exacerbates OGD-induced mitophagy impairment and neuronal apoptosis. (**A**) Representative immunofluorescence images showing Tomm20 (mitochondria, red), LC3B (autophagosomes, green), and merged signals (yellow indicates co-localization). Scale bar: 20 µm. (**B**) Quantification of co-localization (Pearson’s coefficient; *** *p* < 0.001; Data = mean ± SD; *n* = 3 repetitions/group). (**C**) Representative blots of p62, Tomm20, Beclin1, and LC3-I/II. (**D**–**G**) Quantification of protein levels (* *p* < 0.05, ** *p* < 0.01, *** *p* < 0.001; Data = mean ± SD; *n* = 3 repetitions/group). (**H**) Representative blots of Bcl2, Bax, and cleaved caspase-3. (**I**–**K**) Quantification of apoptotic markers (* *p* < 0.05, ** *p* < 0.01, *** *p* < 0.001; Data = mean ± SD; *n* = 3 repetitions/group).

To assess the occurrence of mitophagy, Tomm20/LC3B colocalization analysis was performed. Immunofluorescence revealed that BNIP3 silencing alone did not affect basal mitophagy in normoxic cells, whereas the co-localization of Tomm20 and LC3B was increased in HT22 cells under OGD conditions. However, the colocalization of Tomm20 and LC3B was significantly impaired after silencing BNIP3 (Figure 4A,B).

WB analysis revealed two distinct mechanisms of impaired autophagy. SiBNIP3 treatment alone did not alter the changes in autophagy proteins, while OGD treatment resulted in significant accumulation of p62 and Tomm20, and decreased Beclin1 expression. After silencing BNIP3, this phenomenon was further aggravated, suggesting that the autophagic flow was impaired and autophagy formation was defective. Intriguingly, the LC3-II/LC3-I ratio was significantly increased after OGD treatment, indicating an alteration in autophagic activity, while this ratio was markedly decreased following siBNIP3 treatment. These dynamic changes in LC3-II levels, coupled with the concurrent accumulation of p62 (Figure 4C–G), suggest that both OGD stress and BNIP3 silencing disrupt the flux of mitophagy, albeit potentially through different nodes of the pathway.

Intriguingly, while BNIP3 knockdown alone showed no pro-apoptotic effects, OGD treatment triggered apoptosis, characterized by downregulation of anti-apoptotic Bcl2, upregulation of pro-apoptotic Bax, and cleaved caspase-3. BNIP3 silencing exacerbated these alterations, linking BNIP3 deficiency to synergistic aggravation of both autophagic failure and programmed cell death in ischemic injury (Figure 4H–K).

In conclusion, BNIP3 is essential for activating protective mitophagy in response to OGD stress. Its deficiency leads to a dual defect: impaired clearance of damaged mitochondria and exacerbated activation of the apoptotic pathway, highlighting its critical role in cell fate during ischemic injury.

### 3.6. BNIP3 Overexpression Rescues Mitophagic Flux and Attenuates Apoptosis in OGD Injury

To delineate BNIP3’s dual role in autophagy regulation and apoptosis suppression, we performed gain-of-function studies under OGD conditions. Mitophagy is a multi-step process encompassing autophagosome initiation through lysosomal degradation. To dissect the role of specific nodes within this pathway, we employed two mechanistically distinct autophagy inhibitors—3-MA (early-stage inhibitor) and bafilomycin A1 (BafA1, late-stage inhibitor)—in the OGD model. Compared with the OGD group, BNIP3 overexpression significantly reduced the LC3-II/LC3-I ratio, decreased p62 accumulation, and lowered the level of cleaved caspase-3 (Figure S2A–D). In the presence of 3-MA, the LC3-II/LC3-I ratio was markedly diminished, while p62 accumulated robustly. By contrast, BafA1 treatment substantially increased the LC3-II/LC3-I ratio, yet also induced pronounced p62 accumulation, indicative of a blockade in autophagic flux. Both inhibitors completely abolished the anti-apoptotic effect of BNIP3 overexpression, as evidenced by a significant elevation in cleaved caspase-3 levels (Figure S2A–D). These data demonstrate that although 3-MA and BafA1 impair different stages of autophagy (initiation vs. degradation), both converge on disrupting complete autophagic flux and thereby exacerbate OGD-induced apoptosis. The results confirm that the neuroprotection conferred by BNIP3 is dependent on a functional autophagic–lysosomal pathway.

**Figure 5:**
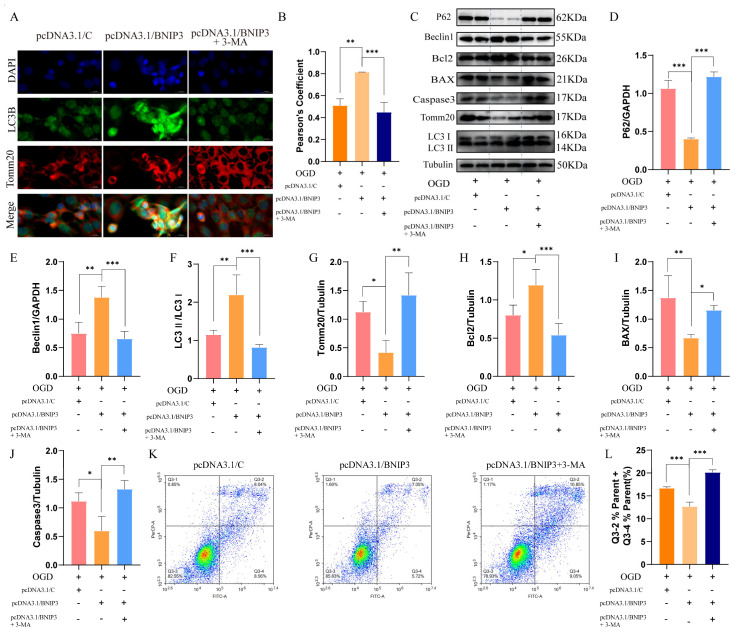
BNIP3 overexpression confers neuroprotection through autophagy-dependent apoptosis suppression. (**A**) Representative immunofluorescence images (LC3B: green; Tomm20: red; co-localization: yellow). Scale bar: 20 μm. (**B**) Quantification of Pearson’s coefficient (** *p* < 0.01, *** *p* < 0.001; Data = mean ± SD; *n* = 3 repetitions/group). (**C**–**J**) WB analysis of autophagy markers and apoptotic pathway (* *p* < 0.05, ** *p* < 0.01, *** *p* < 0.001; Data = mean ± SD; *n* = 4 repetitions/group). (**K**) Annexin V-FITC/PI staining (Q2 + Q4: apoptotic cells). (**L**) Quantification of apoptosis rate (*** *p* < 0.001; Data = mean ± SD; *n* = 3 repetitions/group).

Immunofluorescence analysis revealed that BNIP3 overexpression enhanced mitophagic clearance (increased LC3B-Tomm20 co-localization; Figure 5A,B), and this effect was abolished by 3-MA treatment (Figure 5A,B).

WB analysis revealed coordinated improvements in autophagic flux (reduced p62 and Tomm20 accumulation; Figure 5C,D,G) and enhanced autophagosome biogenesis (elevated Beclin1 and LC3-II/LC3-I ratio; Figure 5E,F) after BNIP3 overexpression. BNIP3 overexpression also upregulated the anti-apoptotic protein Bcl2 (Figure 5H) while downregulating pro-apoptotic Bax and cleaved caspase-3 (Figure 5I,J). Critically, 3-MA treatment reversed all protective effects, demonstrating that BNIP3-mediated apoptosis resistance requires functional autophagy.

Flow cytometric analysis of apoptosis revealed that BNIP3 overexpression conferred significant cytoprotection in OGD-treated cells, reducing the apoptotic cell percentage versus OGD controls (Figure 5K,L). Conversely, pharmacological autophagy inhibition with 3-MA not only abolished this protection but further exacerbated apoptosis compared to OGD alone, confirming that intact complete autophagic flux is essential for BNIP3-mediated neuroprotection. Together, these findings establish that BNIP3 exerts neuroprotective effects against OGD-induced injury through an autophagy-dependent pathway that suppresses apoptotic cell death.

In conclusion, BNIP3 overexpression confers neuroprotection against OGD-induced injury by promoting mitophagic flux, which in turn is essential for attenuating apoptotic cell death. This establishes a direct, autophagy-dependent pathway through which BNIP3 safeguards cells during ischemic stress.

### 3.7. BNIP3L Overexpression Attenuates OGD-Induced Mitochondrial Dysfunction

**Figure 6:**
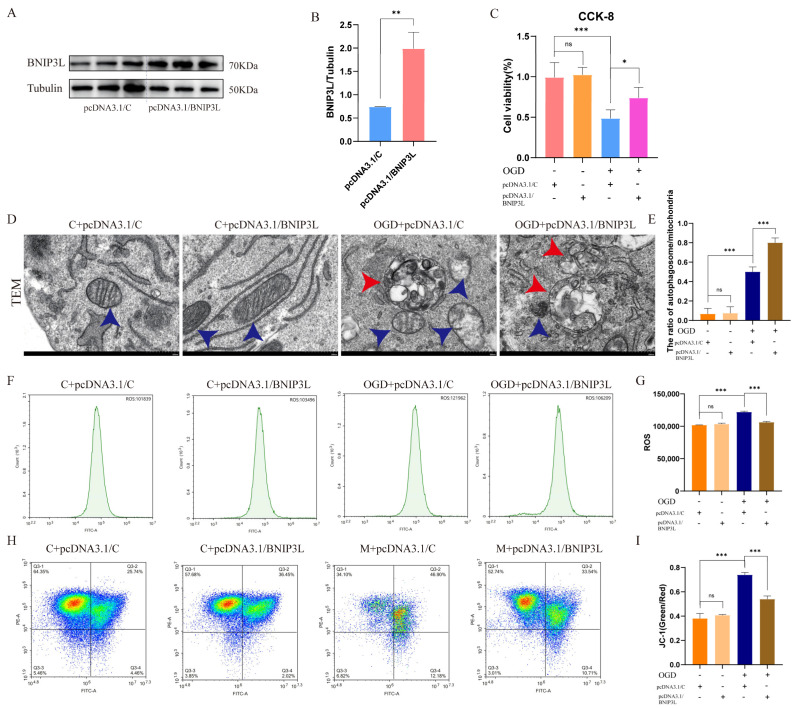
BNIP3L overexpression alleviates OGD-induced neuronal damage by promoting mitophagy and mitochondrial function. (**A**) Representative WB of BNIP3L after pcDN13.1 transfection. (**B**) Quantification of BNIP3L protein levels (** *p* < 0.01; Data = mean ± SD; *n* = 3 repetitions/group). (**C**) Cell viability (CCK-8 assay) in HT22 neurons under OGD with/without pcDNA3.1/BNIP3L (* *p* < 0.05, *** *p* < 0.001; Data = mean ± SD; *n* = 3 repetitions/group). (**D**) Representative TEM images of each group in HT22 cells (scale bar: 500 nm). (**E**) The ratio of autophagic structures to mitochondria across multiple fields from each group (*** *p* < 0.001, *n* = 3 repetitions /group). (**F**) Representative flow cytometry images of each group. (**G**) Quantification of ROS intensity (*** *p* < 0.001; Data = mean ± SD; *n* = 3 repetitions/group). (**H**) JC-1 aggregates (red, healthy) and monomers (green, depolarized). (**I**) Quantification of JC-1 green/red ratio (*** *p* < 0.001; Data = mean ± SD; *n* = 3 repetitions/group).

To establish a causal relationship between BNIP3L and mitochondrial quality control, we engineered HT22 cells with stable BNIP3L overexpression (>70% transfection efficiency validated by WB; Figure 6A,B). Functional assays revealed that BNIP3L overexpression conferred robust cytoprotection in OGD-treated HT22 cells. The CCK-8 assay showed a significant improvement in cell viability versus OGD controls, with no toxicity under normoxia (Figure 6C).

TEM revealed that under normoxic conditions, mitochondrial structure remained intact with clearly visible cristae. Following OGD treatment, mitochondrial structure became disrupted, showing swelling and loss of cristae, accompanied by the formation of mitophagosomes. Overexpression of BNIP3L significantly reduced the number of damaged mitochondria, promoted the formation of numerous mitophagosomes, and markedly increased the autophagosome-to-mitochondria ratio (Figure 6D,E).

Parallel measurements demonstrated that BNIP3L overexpression reduced OGD-induced ROS accumulation (Figure 6F,G) and restored mitochondrial membrane potential (Figure 6H,I).

These findings collectively establish that BNIP3L overexpression preserves neuronal viability during ischemic insult by mitigating oxidative stress and stabilizing mitochondrial function.

### 3.8. BNIP3L Overexpression Enhances Mitophagic Flux and Attenuates OGD Injury

**Figure 7:**
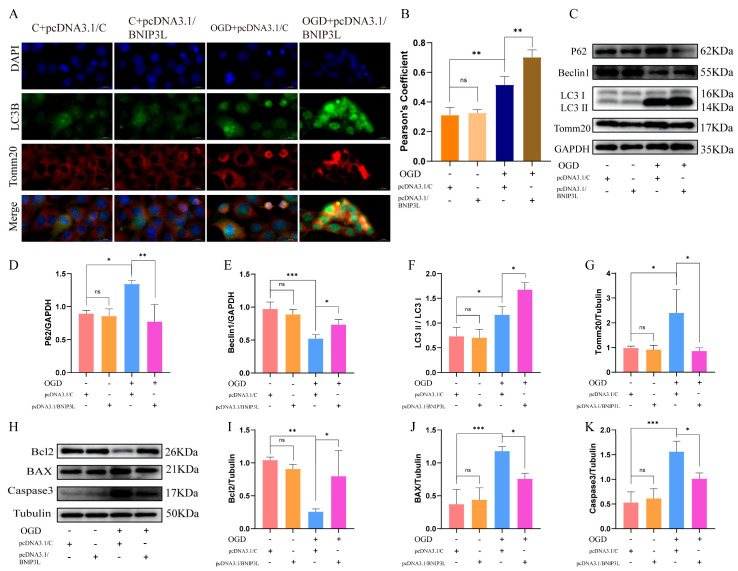
BNIP3L overexpression alleviates OGD-induced mitophagy damage and neuronal apoptosis. (**A**) Representative immunofluorescence images showing Tomm20 (mitochondria, red), LC3B (autophagosomes, green), and merged signals (yellow indicates co-localization). Scale bar: 20 µm. (**B**) Quantification of co-localization (Pearson’s coefficient; ** *p* < 0.01; Data = mean ± SD; *n* = 3 repetitions/group). (**C**) Representative blots of p62, Tomm20, Beclin1, and LC3-I/II. (**D**–**G**) Quantification of protein levels (* *p* < 0.05, ** *p* < 0.01, *** *p* < 0.001; Data = mean ± SD; *n* = 3 repetitions/group). (**H**) Representative blots of Bcl2, Bax, and cleaved caspase-3. (**I**–**K**) Quantification of apoptotic markers (* *p* < 0.05, ** *p* < 0.01, *** *p* < 0.001; Data = mean ± SD; *n* = 3 repetitions/group).

Immunofluorescence analysis demonstrated that BNIP3L overexpression significantly enhanced mitochondrial clearance, as evidenced by a remarkable increase in LC3B-Tomm20 co-localization compared to vector controls in OGD (Figure 7A,B). WB analysis revealed coordinated improvements in multiple aspects of autophagic function (Figure 7C–G). Markers of autophagic flux showed significant improvement, with p62 levels reduced (Figure 7D) and mitochondrial protein Tomm20 decreased (Figure 7G). Key regulators of autophagosome formation were upregulated, including Beclin1 (Figure 7E) and the LC3-II/LC3-I ratio (Figure 7F).

Importantly, BNIP3L overexpression exerted significant neuroprotective effects in the OGD model. No cytotoxic effects were observed under normoxic conditions (Figure 7H–K). In OGD-treated cells, BNIP3L overexpression significantly attenuated apoptotic activation (Figure 7H–K), as indicated by increased Bcl2 protein expression and decreased expression of the pro-apoptotic proteins BAX and cleaved caspase-3.

These findings establish that BNIP3L-mediated enhancement of mitophagic flux plays a crucial role in protecting neurons against ischemic injury through coordinated improvement of mitochondrial quality control and suppression of apoptotic pathways.

### 3.9. BNIP3L Overexpression Enhances Autophagy-Dependent Neuroprotection in OGD Injury

**Figure 8:**
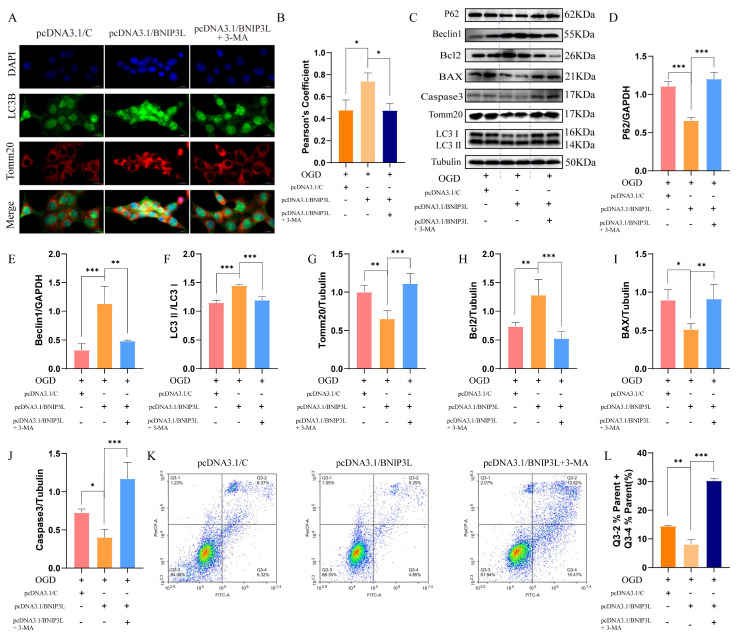
BNIP3L overexpression confers neuroprotection through autophagy-dependent apoptosis suppression. (**A**) Representative immunofluorescence images (LC3B: green; Tomm20: red; co-localization: yellow). Scale bar: 20 μm. (**B**) Quantification of Pearson’s coefficient (* *p* < 0.05; Data = mean ± SD; *n* = 3 repetitions/group). (**C**–**J**) WB analysis of autophagy markers and apoptotic pathway (* *p* < 0.05, ** *p* < 0.01, *** *p* < 0.001; Data = mean ± SD; *n* = 4 repetitions/group). (**K**) Annexin V-FITC/PI staining (Q2 + Q4: apoptotic cells). (**L**) Quantification of apoptosis rate (** *p* < 0.01; *** *p* < 0.001; Data = mean ± SD; *n* = 3 repetitions/group).

To elucidate BNIP3L’s dual regulatory role in mitochondrial quality control and apoptosis, we conducted systematic gain-of-function studies in HT22 neurons. Immunofluorescence analysis revealed that BNIP3L overexpression enhanced mitophagic flux (Figure 8A,B). However, 3-MA treatment abolished this enhancement (Figure 8A,B).

WB analysis demonstrated coordinated improvements across the autophagy cascade (Figure 8C–G), including autophagic flux restoration (decreased expression of P62 and Tomm20) and autophagosome biogenesis activation (increased expression of Beclin1 and increased LC3-II/LC3-I ratio). Concurrently, BNIP3L overexpression shifted the apoptotic balance toward survival (increased expression of Bcl2 and decreased expression of BAX and Caspase3) (Figure 8H–J). However, 3-MA treatment reversed the trend.

Quantitative flow cytometry demonstrated that BNIP3L overexpression provided robust protection against OGD-induced apoptosis (Figure 8K,L). The autophagy inhibitor 3-MA completely reversed this protective effect.

These findings establish that BNIP3L-mediated neuroprotection requires functional complete autophagic-lysosomal pathway, as its anti-apoptotic effects were entirely dependent on intact autophagy pathways.

## 4. Discussion

VD, the second most prevalent form of dementia globally, remains poorly understood mechanistically despite its strong association with CCH-induced mitochondrial dysfunction [34,35]. Mitophagy—the selective clearance of damaged mitochondria via autophagic-lysosomal degradation—plays a neuroprotective role by preserving neuronal viability and supporting cognitive recovery [36]. While mitophagy has emerged as a critical quality control mechanism in neurodegenerative diseases, its role in VD pathogenesis—particularly under chronic ischemic conditions—has been underexplored. Our study bridges this gap by systematically investigating BNIP3/BNIP3L-mediated mitophagy in both CCH models and OGD systems, revealing three groundbreaking insights with translational implications.

Firstly, BNIP3/BNIP3L are central regulators of mitophagy in chronic ischemic diseases. Contrary to the prevailing paradigm emphasizing PINK1-Parkin dominance in acute mitochondrial stress [37,38,39], we demonstrate that receptor-mediated mitophagy via BNIP3/BNIP3L is indispensable under chronic hypoperfusion. The unchanged PINK1/Parkin levels, coupled with FUNDC1 upregulation, suggest a compensatory mechanism distinct from acute injury models. This aligns with emerging evidence that FUNDC1 coordinates hypoxia adaptation [40] but diverges by revealing BNIP3/BNIP3L suppression as the primary defect in CCH. Our finding that BNIP3/BNIP3L silencing reduced mitophagic flux challenges the view that multiple mitophagy pathways are functionally redundant [41,42,43]. Previous studies have shown that the deficiency of hypoxia-induced mitochondrial autophagy receptors BNIP3/BNIP3L also increases hypoxia-induced cell death [44,45], and the activation of BNIP3/BNIP3L can restore mitochondrial autophagy in the absence of the Pink1-Parkin-mediated pathway [46,47]. In our study, the absence of BNIP3/BNIP3L further aggravated neuronal apoptosis caused by autophagic absence, while the activation of BNIP3/BNIP3L reversed mitochondrial autophagy and neuronal apoptosis, which was consistent with the above studies.

Secondly, there is a dual modulation of autophagy-apoptosis crosstalk in CCH. Our data delineate a clear pathological sequence in VD: chronic ischemia primarily suppresses the expression of the mitophagy receptors BNIP3 and BNIP3L, as evidenced by their marked downregulation in both in vivo and in vitro models. This suppression of receptor abundance constitutes the upstream defect, which in turn impairs the activation of receptor-mediated mitophagy—specifically, the recruitment of autophagosomes to damaged mitochondria. Consequently, the compromised clearance of dysfunctional mitochondria leads to ROS accumulation and apoptosis. Importantly, our gain-of-function experiments demonstrate that restoring BNIP3/BNIP3L expression is sufficient to rescue mitophagic flux and neuronal survival, indicating that the signaling capacity of this pathway remains intact but is pathologically muted. Therefore, therapeutic strategies for VD may need to focus on counteracting this suppression to reinstate mitochondrial quality control. The reduction in apoptosis with BNIP3/BNIP3L overexpression was substantially reversed by 3-MA, supporting a critical autophagy-dependent component in the anti-apoptotic mechanism of BNIP3/BNIP3L. This is consistent with reports of direct anti-apoptotic effects of BNIP3/BNIP3L in other disease models [47,48,49,50,51], highlighting the ubiquity of autophagy and apoptosis interactions. The autophagy-dependent modulation of Bcl2/Bax suggests a plausible mechanism whereby mitophagic clearance of damaged mitochondria prevents cytochrome c leakage, thereby inhibiting caspase-3 activation. This proposed mechanism, however, remains a hypothesis to be tested in future studies specifically designed to assess cytochrome c release.

Thirdly, this study highlights the therapeutic implications of VD management. Our discovery that BNIP3/BNIP3L overexpression restores ΔΨm and reduces ROS identifies these receptors as promising therapeutic targets. The attenuation of oxidative stress (ROS reduction) observed upon BNIP3/BNIP3L overexpression is likely an indirect, yet critical, outcome of enhanced mitophagic flux. As dedicated receptor proteins, their primary function is to target depolarized and damaged mitochondria for autophagic clearance. By efficiently removing these dysfunctional organelles—which are major sources of aberrant ROS production—BNIP3/BNIP3L help to restore the redox balance of the neuronal mitochondrial network. This mechanism is consistent with our finding that their protective effects, including ROS reduction, are fully dependent on an intact autophagic-lysosomal pathway, as inhibition by either 3-MA or bafilomycin A1 completely reversed the benefits. The aggravated pathology with Mdivi-1 further cautions against broad-spectrum mitophagy inhibitors in chronic cerebrovascular diseases. This provides us with new targets and therapeutic opportunities for the prevention and treatment of VD.

The central role of BNIP3 and BNIP3L in orchestrating mitochondrial homeostasis under chronic hypoxia extends far beyond their canonical function as mitophagy receptors. Our findings illuminate their multidimensional regulatory capacity at the intersection of redox equilibrium and energy adaptation—a nexus critical for neuronal survival in VD. BNIP3/BNIP3L-mediated mitophagy serves as a first-line defense against oxidative catastrophe in CCH. By selectively eliminating depolarized mitochondria, these receptors prevent the vicious cycle of ROS-induced ROS release (RIRR) that amplifies oxidative damage. Similarly, other studies have also demonstrated the crucial role of BNIP3/ BNIP3L-dependent mitochondrial autophagy in clarifying ROS [52,53,54,55,56]. Notably, in the OGD model of the present study, we found that LC3-II was significantly increased, which may be related to the activation of autophagic vesicles caused by the large accumulation of ROS [55,56,57].

Our research still has some limitations. Firstly, although our rodent model successfully summarized the key characteristics of VD (for example, chronic perfusion insufficiency, cognitive decline), the key differences between the cerebrovascular systems of rats and humans need to be handled with caution in direct transformation. Rodents exhibited higher baseline cerebral blood flow and unique white matter structure [58,59]. To solve this problem, we can give priority to using multiple immunohistochemistry techniques to conduct autopsy analysis on the hippocampus of VD patients to determine the changes in the BNIP3/BNIP3L axis. Or generate 3D vascularized organoids from IPscs of VD patients to test BNIP3/ BNIP3L targeted intervention. Secondly, the present study employed a homogeneous neuronal cell line (HT22) to delineate cell-intrinsic mechanisms. While this approach was essential for establishing a clear causal link between BNIP3/BNIP3L suppression, mitophagic failure, and neuronal apoptosis, it does not capture the potential contributions of other cell types within the neurovascular unit (e.g., endothelial cells, pericytes) to the overall pathogenesis of VD. Future studies investigating the role of BNIP3/BNIP3L in these vascular cells and their interplay with neurons are warranted. Thirdly, the partial preservation of mitophagy in BNIP3/BNIP3L-silenced cells suggests unexplored compensatory mechanisms. Crispr-cas9-mediated BNIP3/BNIP3L double KO may provide assistance in revealing the potential pathways and crosstalk in future studies [60]. Furthermore, our in vitro findings were established in HT22 cells, which lack functional ionotropic glutamate receptors. While this allowed for a focused investigation into the neuron-intrinsic mitophagic response to direct metabolic stress (OGD), it does not model receptor-mediated excitotoxicity. Future studies employing primary hippocampal neurons or glial-neuronal co-cultures will be important to validate the protective role of BNIP3/BNIP3L against excitotoxic insults that more fully mimic one aspect of the complex VD pathology. Additionally, the in vitro findings are based on a single, sustained OGD duration (24 h). Future studies employing milder or shorter OGD exposures, or a detailed time-course analysis, would be valuable to establish the precise temporal relationship between BNIP3/BNIP3L downregulation, the onset of mitophagic impairment, and neuronal injury progression.

While our study provides the first mechanistic atlas of BNIP3/BNIP3L in CCH, these limitations chart a clear path for next-generation VD research. Addressing these gaps will accelerate the development of precision mitophagy therapies tailored to disease stage, sex, and vascular risk profile.

## 5. Conclusions

This study establishes BNIP3 and BNIP3L as critical regulators of mitochondrial quality control in VaD, demonstrating their essential role in maintaining neuronal survival under CCH. Importantly, this work redefines the mechanistic understanding of VD, identifying BNIP3 and BNIP3L as central players in chronic ischemic neuroprotection. Their dual role in mitophagy regulation and apoptosis suppression offers novel therapeutic avenues for mitigating vascular cognitive decline. Future studies should focus on translating these findings into clinically viable strategies for VD treatment.

## Data Availability

Data will be made available upon request.

## Supplementary Materials

The following supporting information can be downloaded at: https://www.mdpi.com/article/10.3390/cells15070585/s1, Figure S1: In the CCH model, PINK1 and Parkin expression levels remained unchanged, whereas FUNDC1 was significantly upregulated; Figure S2: Effects of 3-MA and Bafilomycin A1 on BNIP3 overexpression in the OGD model.

## Abbreviations

The following abbreviations are used in this manuscript:

VD	Vascular dementia
BCCAO	Bilateral common carotid artery occlusion
OGD	Oxygen–glucose deprivation
ROS	Reactive oxygen species
AD	Alzheimer’s disease
CCH	Chronic cerebral hypoperfusion
OMM	Outer mitochondrial membrane
CBF	Cerebral blood flow
MWM	Morris water maze
TEM	Transmission electron microscope
MMP	Mitochondrial membrane potential
