# Supplementary material for: BNIP3/BNIP3L-Dependent Mitophagy Protects Against Hippocampal Neuronal Damage and Apoptosis in a Model of Vascular Dementia
Source: Cells. 2026 Mar 25;15(7):585. doi: 10.3390/cells15070585 (PMC13071978; doi:10.3390/cells15070585)
Supplement: Supplementary file 1 [file cells-15-00585-s001.zip › cells-4102799-supplementary.pdf]

Figure S1

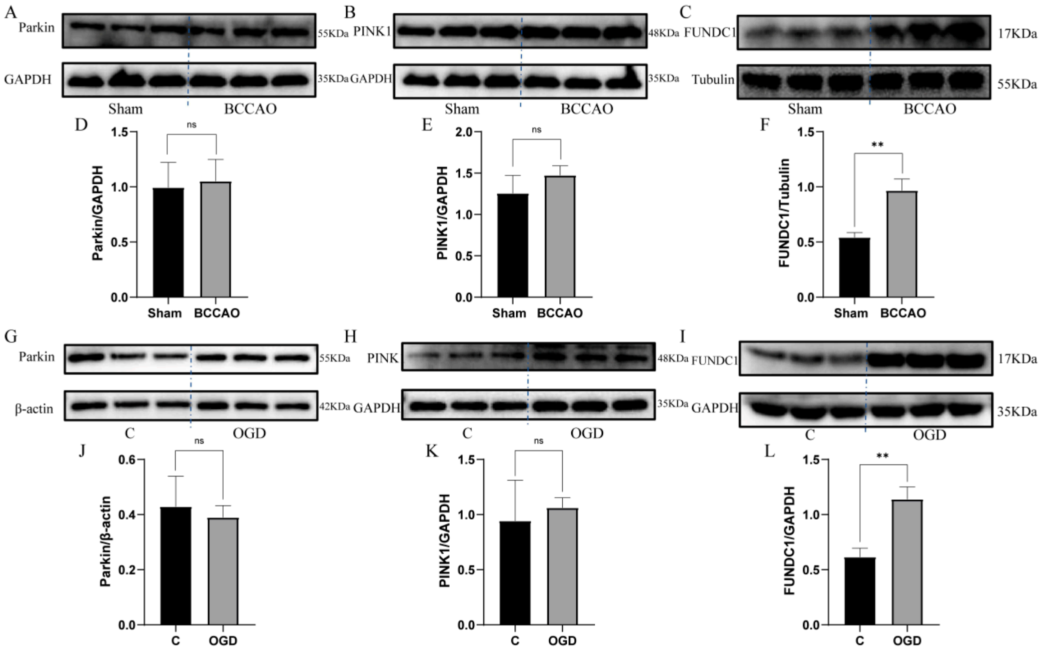

Figure S1. In the CCH model, PINK1 and Parkin expression levels remained unchanged, whereas FUNDC1 was significantly upregulated. (A, D) WB bands and quantitative analysis of PINK1 in the Sham and BCCAO groups (in vivo models; Data = mean ± SD; n = 3 repetitions/group).

(B, E) WB bands and quantitative analysis of Parkin expression in the Sham and BCCAO groups (in vivo models; Data = mean ± SD; n = 3 repetitions/group).

(C, F) WB bands and quantitative analysis of FUNDC1 expression in the Sham and BCCAO groups (in vivo models; $**p < 0.01$; Data = mean ± SD; n = 3 repetitions/group).

(G, J) WB bands and quantitative analysis of Parkin expression in the Control and OGD groups (in vitro models; Data = mean ± SD; n = 3 repetitions/group).

(H, K) WB bands and quantitative analysis of PINK1 expression in the Control and OGD groups (in vitro models; Data = mean ± SD; n = 3 repetitions/group).

(I, L) WB bands and quantitative analysis of FUNDC1 expression in the Control and OGD groups (in vitro models; $**p < 0.01$; Data = mean ± SD; n = 3 repetitions/group).

Figure S2

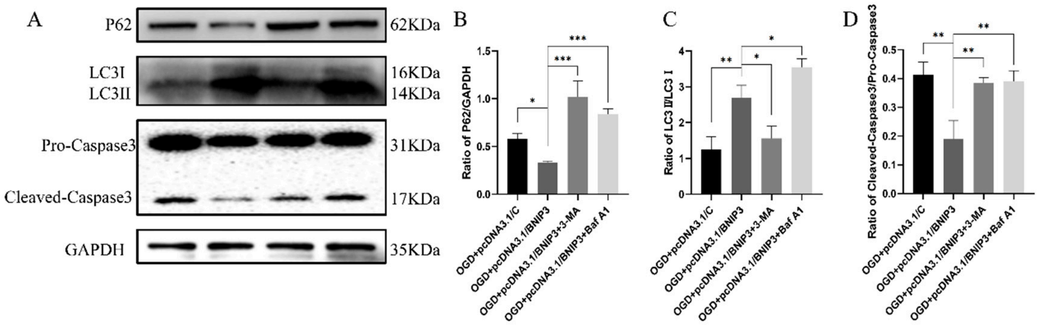

Figure S2. Effects of 3-MA and Bafilomycin A1 on BNIP3 overexpression in the OGD model. (A) Representative WB images showing the expression of P62, LC3B, and Cleaved Caspase3 under OGD conditions with BNIP3 overexpression, and following co-treatment with 3-MA or Bafilomycin A1 (BafA1). (B) Quantification of P62 protein levels. (C) Quantification of the LC3-II/LC3-I ratio. (D) Quantification of Cleaved Caspase3 protein levels. Data are presented as mean ± SD; n = 3 independent replicates. Statistical analysis was performed using one-way ANOVA. *$p < 0.05$, **$p < 0.01$, ***$p < 0.001$ vs.
